# Herpes Simplex Virus Type 1 Enhances Expression of the Synaptic Protein Arc for Its Own Benefit

**DOI:** 10.3389/fncel.2018.00505

**Published:** 2019-01-08

**Authors:** Francisca Acuña-Hinrichsen, Mariela Muñoz, Melissa Hott, Carolina Martin, Evelyn Mancilla, Paula Salazar, Luis Leyton, Angara Zambrano, Margarita I. Concha, Patricia V. Burgos, Carola Otth

**Affiliations:** ^1^Institute of Clinical Microbiology, Faculty of Medicine, Universidad Austral de Chile, Valdivia, Chile; ^2^Centro de Excelencia en Estudios Morfológicos y Quirúrgicos (CEMyQ), Universidad de La Frontera, Temuco, Chile; ^3^Center for Interdisciplinary Studies on the Nervous System (CISNe), Universidad Austral de Chile, Valdivia, Chile; ^4^Institute of Biochemistry and Microbiology, Faculty of Science, Universidad Austral de Chile, Valdivia, Chile; ^5^Institute of Physiology, Faculty of Medicine, Universidad Austral de Chile, Valdivia, Chile; ^6^Centro de Biología Celular y Biomedicina, Facultad de Ciencia y Facultad de Medicina, Universidad San Sebastián, Santiago, Chile; ^7^Center for Aging and Regeneration (CARE), Facultad de Ciencias Biológicas, Pontificia Universidad Católica de Chile, Santiago, Chile

**Keywords:** HSV-1, neuronal dysfunction, Arc, neurodegeneration, neuronal infection, Alzheimer’s disease, neurotropic virus

## Abstract

Herpes simplex virus type 1 (HSV-1) is a neurotropic virus able to reach the central nervous system (CNS) after primary infection in oronasal mucosa. HSV-1 establishes latency inside neurons due the repression of its gene expression process, which is related to periodic reactivations in response to cellular stress conditions, constituting a risk factor for neurodegenerative diseases such as Alzheimer’s disease (AD). The immediate-early gene Arc plays an essential role in neuronal morphology, synaptic plasticity and memory formation. Arc acts as a hub protein, interacting with components of the endocytic machinery required for AMPA receptor (AMPAR) recycling as well as with proteins of the post-synaptic density and actin cytoskeleton. However, to date, no studies have evaluated whether persistent neurotropic HSV-1 infection modulates the expression or function of Arc protein in brain tissue. Here, we report that neuronal *in vivo* and *in vitro* infection of HSV-1 significantly increases Arc protein levels, showing a robust perinuclear distribution in neuronal cell lines, a process that is dependent on an active HSV-1 replication cycle. Finally, we found that silencing Arc protein caused a decrease in HSV-1 proteins and viral progeny, suggesting that Arc is involved in the lifecycle of HSV-1. Our studies strongly suggest that pathogenicity of HSV-1 neuronal reactivations in humans could be mediated in part by Arc neuronal upregulation and its potential role in endocytic trafficking and AMPA-neuronal function impairment. Further studies are necessary to define whether this phenomenon could have repercussions in cognition and learning processes in infected individuals.

## Introduction

Herpes simplex virus type 1 (HSV-1) is a ubiquitous and neurotropic virus associated with cold sores that affects close to 68% of the adult population worldwide. However, only 20%–40% of infected individuals develop evident clinical symptomatology (Schillinger et al., 2004; Diefenbach et al., 2008). HSV-1 establishes and maintains a lifelong latent infection in peripheral neurons with frequent reactivation episodes (Davidovici et al., 2006; Xu et al., 2006). Once the viral genome enters the neuronal nucleus, one of two processes begins either productive replication or repression of lithic genes, leading to the establishment of latency (Ortiz et al., 2003). An exciting issue is whether recurrent viral reactivations at the neuronal level could cause neuronal damage or neurodysfunction repeatedly (Ando et al., 2008; Conrady et al., 2010; Martin et al., 2014a; Harris and Harris, 2015; Otth et al., 2016). Nevertheless, it is still unknown whether neuronal HSV-1 infection could have an impact on synaptic plasticity.

Synaptic plasticity is defined as a change in the strength of synaptic connections induced by experience (Bliss and Collingridge, 1993). It has been known that long-lasting forms of synaptic plasticity such as long term potentiation (LTP) are dependent on rapid, *de novo* RNA and protein synthesis, while short-term plasticity forms are not (Okuno, 2011). Thus, gene expression occurring immediately after the events to be memorized appears to play a critical role in establishing and/or maintenance of long-lasting neuronal changes. One of the most important immediate-early genes (IEGs) related to synaptic plasticity processes is the master regulator of plasticity protein Arc (Activity-Regulated Cytoskeleton-associated protein, also known as Arg3.1), which has emerged as a key protein in memory formation and diverse types of synaptic plasticity including LTP and long term depression (LTD) as well as synaptic scaling (Bramham et al., 2010; Siddoway et al., 2014; Qiu et al., 2016).

Arc mRNA is rapidly transported to distal dendrites and selectively localizes at activated synapses, where it has the potential to be locally translated (Steward and Worley, 2001; Bramham, 2007; Soulé et al., 2012), followed by rapid protein degradation. Arc regulates dendritic spine density and morphology since it induces changes in cytoskeleton dynamics as a result of its interactions with post-synaptic density proteins (Messaoudi et al., 2007; Peebles et al., 2010). Arc also interacts with components of the clathrin-mediated endocytosis, such as endophilin-3 and dynamin-2, to promote internalization of AMPA receptors (AMPARs) and LTP (Chowdhury et al., 2006; Shepherd et al., 2006; Bramham et al., 2010). Structurally, Arc is a flexible modular protein that contains two structured regions flanking a central hinge domain that confers the ability of reversible self-oligomerization (Myrum et al., 2015; Zhang et al., 2015). Arc emerges as a multifunctional activity-induced hub protein with a central role in long-term synaptic plasticity (Nikolaienko et al., 2017).

Several research groups, including ours, have been working on elucidating a possible relationship between HSV-1 neuronal infection and aged neurodegenerative processes (Zambrano et al., 2008; De Chiara et al., 2010; Piacentini et al., 2015; Otth et al., 2016; Itzhaki and Tabet, 2017). In *in vitro* and *in vivo* assays, we have found that HSV-1 induced significant modifications of the microtubular dynamics together with damage in axonal and dendritic processes (Zambrano et al., 2008; Lerchundi et al., 2011). Furthermore, HSV-1 neuronal infection increased Tau modifications that are associated to neurodegenerative diseases (Zambrano et al., 2008; Lerchundi et al., 2011; Martin et al., 2014b; Leyton et al., 2015; Otth et al., 2016). The main focus of this work was to study the effect of *in vivo* and *in vitro* HSV-1 infection on the expression and distribution of Arc in brain cells and tissue. Here, we report for the first time a relationship between HSV-1 neuronal infection and Arc protein dysregulation, with an impact on viral progeny and AMPA-neuronal function impairment.

## Materials and Methods

### Biosafety Methodology

We used about 20 pregnant mice per year, (considering minimal use of animal to validate results), provided by the Unit of Mice Breeding at the Department of Immunology. In this Unit animals are kept under standard conditions (temperature, feeding, light and water). Trained personnel sacrificed mice using lethal doses of intravenous sodium pentabarbitone (200 mg/kg of total weight). Death was confirmed observing cessation of heartbeat and respiration, and absence of reflexes, in agreement with international standards (www.lal.org.uk). Our laboratory has the appropriate necessary procedures and biosafety equipment for biological elements and chemical disposal in accordance with the Safety Manual of Procedures and Handling of Wastes from the Universidad Austral de Chile, and Biosafety Regulations from CONICYT. Moreover, biosafety measures and containment barriers recommended by CONICYT and WHO for type II risk organisms are routinely used. Animal handling was according to the Experimentation Animals Regulations from the Universidad Austral de Chile, the protocol was approved by the Bioethical Committee of the Austral University of Chile. The Laboratory of Molecular Virology is equipped with a level 2 biosafety chamber, where we carried out HSV-1 propagation and infection experiments. All the activities were authorized and supervised by the principal investigator.

### Cell Culture

The H4 (Human neuroglioma ATCC^®^HTB-148™; Manassas, VA, USA), SH-SY5Y (Human neuroblastoma ATCC^®^ CRL-2266™; Manassas, VA, USA), and HT22 (Murine hippocampal neuronal cell line was kindly supplied by Dr. David Schubert, Salk Institute, San Diego, USA) cell lines were maintained in Dulbecco’s modified Eagle’s medium (DMEM) supplemented with 10% fetal bovine serum (FBS, Invitrogen, Carlsbad, CA, USA) and penicillin/streptomycin (Thermo Fisher Scientific, Waltham, MA, USA) in a 5% CO_2_ atmosphere at 37°C. HT22 and SH-SY5Y were differentiated using Neurobasal media (Gibco, Life Technology, NY, USA) containing N2 supplement and 2 mM L-glutamine (Gibco, Life Technology, NY, USA), as well as DMEM F12 supplemented with 5 μM retinoic acid and 2% of FBS (see controls in Supplementary Figure S1) for 2 and 5 days prior to infection with HSV-1, respectively.

Primary cortical neuronal culture was established with 16-day-old mice embryos (E16). The animals were sacrificed by lethal doses of intravenous sodium pentabarbitone (200 mg/kg of total weight). Death was confirmed by observing cessation of heartbeat and respiration along with an absence of reflexes, in agreement with international standards^1^. Briefly, embryos (E16) were removed from the mice and cortex pairs were dissected. All the tissues were collected in a conical tube containing Hibernate-E complete medium (Gibco, Life Technology, NY, USA) supplemented with 200 mM L-glutamine and B-27 50X. The tissue was treated with 0.25% trypsin-EDTA at 37°C for 5 min and then disaggregated by mechanical grinding with a sterile, fire-polished glass Pasteur pipette, in DMEM supplemented with 10% FBS. Cells were seeded onto coverslips or in 35-mm plastic dishes pre-coated with 10 μg/ml poly-D-lysine (mol. wt > 350 kDa; Sigma-Aldrich Corporation, St Louis, MO, USA). After 20 min in 5% CO_2_ and 95% air at 37°C, floating cells were removed and attached cells cultured for 12–14 days (12–14 DIV) in Neurobasal Medium (Gibco, Life Technology, NY, USA) supplemented with B27 (Gibco, NY, USA), 100 U/ml penicillin, 100 μg/ml streptomycin, and 0.5 mM L-glutamine (Nalgene, Rochester, NY, USA).

### TCID_50_ Assay Protocol

To determine the amount of infectious viral particles present in the cell extracts, we performed a TCID_50_ assay (Tissue Cultured Infective Dose). Vero cells were plated in a 96-well plate and grown until they reached 80% of confluence. Serial dilutions of the virus sample were made (quadruplicate) and incubated with the Vero cells for 1 h at 37°C. Media were aspirated and fresh DMEM containing 5% FBS was added, incubating the plates at 37°C for 4 days. Subsequently, the medium was removed and cells were incubated with 75% methanol for 15 min and stained with crystal violet for 1 min. The plates were washed with water and the number of wells in which the cell monolayer was detached from each dilution was counted. The titer of the virus stock was expressed as the TCID50, which was calculated using a statistical Excel program. The viral titer (PFU/ml) was achieved by multiplying the factor 0.69 TCID_50_/mL according to the Reed-Muench method (Reed and Muench, 1938).

### *In vitro* HSV-1 Infection

The HSV-1 (strain F) used in this study was kindly supplied by Dr. Bernard Roizman, Northwestern University, Chicago, IL, USA. The virus stocks were prepared and titrated from infected Vero cells (Ejercito et al., 1968). Infection was carried out at a multiplicity of infection (MOI) of 10 for Western blot and RT-qPCR experiments and at a MOI of 5 for immunofluorescence experiments. The virus was allowed to adsorb for 1 h in a low volume of medium supplemented with 2% FBS for cell lines and B27 for primary cultures, with regular mixing. Following infection, the viruses were removed by aspiration, the cells were washed once with PBS and finally fresh normal media were added. HSV-1 and mock-infected cells were cultivated further for different time periods (1, 4, 8 and 24 h post infection: hpi). For the viral replication inhibition experiment, neuronal culture was treated with 50 μM acyclovir (ACV) 24 h before HSV-1 infection, keeping ACV during the entire infection protocol. The efficiency of the inhibition was 45%.

### Immunohistochemistry

Tissue sections of the *in vivo* model of HSV-1 infection previously done by Martin et al. (2014a) were used. In brief, the tissues were deparaffinized with xylene and rehydrated through a series of graded ethanol. Endogenous peroxidase was quenched in a 0.3% (v/v) H_2_O_2_/methanol bath for 5 min followed by several washes with PBS 1× pH 7.4. Slides were incubated for 60 min at room temperature in 5% (w/v) BSA-PBS pH 7.4, followed by incubation overnight at 4°C with primary antibodies (1:100 dilution) in 1% (w/v) BSA-PBS pH 7.4 and 0.3% (v/v) Triton X-100. The antibodies used were anti-Arc (16290-1-AP, Proteintech Europe) and anti-ICP8 (sc-53329, clone 10A3, Santa Cruz Biotechnology, Dallas, TX, USA). Tissues were washed and incubated with biotinylated secondary antibodies (anti-mouse, anti-rabbit and anti-goat IgG), and afterwards with avidin-horseradish peroxidase conjugates using the reagents provided by the manufacturer (Kit LSAB systems K0690, DAKO). Immunostaining was developed using 0.05% (w/v) diaminobenzidine and 0.03% (v/v) H_2_O_2_. Sections incubated without primary antibodies or with preimmune serum were used as controls. The markers were evaluated in the same cortex area (piriform area), a region near the olfactory bulb, which corresponds to the central nervous system (CNS) entry site of HSV-1 during intranasal inoculation. Stained slides were examined with a Zeiss Axioskope II microscope equipped with a digital video camera (NikonDXM1200). The cortex from mock and HSV-1 infected mice [six animals per time: 15, 60 days post-infection (dpi)] was analyzed (three slices per animal). HSV-1 infection was detected in serial cortex slices with a specific antibody against viral protein ICP8 (three slices per animal). The images obtained were processed with Adobe Photoshop 6.0. Positively stained cells were counted on slides obtained from the same cortex region in control and infected animals in an area of 200 μm^2^ of the captured images using the ImageJ program. The mean number of immunoreactive cells for each antibody at different times post-infection was determined in triplicate.

### Fluorescence Microscopy and Antibodies

Non-infected neuronal cells (Mock) and HSV-1-infected neuronal cells were fixed in 4% paraformaldehyde or Methanol in PBS 1X for 30 min, or 2 min, respectively. They were then washed three times in PBS 1× and permeabilized in 0.2% Triton X-100 in PBS 1× for 10 min (only the PFA fixed cells). Cells were incubated for 30 min at 37°C with the following primary antibodies: against HSV-1 ICP8 (sc-53329, clone 10A3, Santa Cruz) or ICP5 (sc-56989, Santa Cruz) viral proteins, Arc (16290-1-AP, Proteintech Europe), and AMPA subunit GluR2 (Ab-31232, Abcam). The same protocol was used with the phalloidin probe to analyze the actin cytoskeleton. Finally, cells were incubated with the corresponding secondary antibodies conjugated with Alexa-488 or Alexa-594, and nuclei were stained with DAPI (Invitrogen, Carlsbad, CA, USA). Fluorescence images were obtained using a Zeiss Axioskope A1 epifluorescence microscope (Carl Zeiss, Göttingen, Germany) with a digital video camera (Nikon DXM 1200). The images obtained (at least five microscopic fields per sample) were processed with ImageJ software (National Institutes of Health).

### Western Blotting

For biochemical analysis, neurons from different treatments were harvested and lysed in RIPA buffer 1× supplemented with protease and phosphatase inhibitors (Sigma-Aldrich Corporation St Louis, MO, USA) and the protein concentration was quantified with a Micro BCA™ Protein Assay Kit (Thermo Fischer Scientific, Waltham, MA, USA). Equal amounts of protein (20 μg per line) were loaded and separated by SDS-PAGE (10% polyacrylamide) and transferred to nitrocellulose membranes (Thermo Fischer Scientific, Waltham, MA, USA). The membranes were incubated at room temperature in blocking solution (3% BSA in TBS-Tween) and then incubated overnight with primary antibodies diluted in TBS-1% BSA: anti-β-actin (PA1-4659, Thermo Fischer Scientific, Waltham, MA, USA), anti-Arc (16290-1-AP, Proteintech Europe), anti-GAPDH (MA5-15738, Thermo Fischer Scientific, Waltham, MA, USA), and anti-ICP8 (sc-53329, clone 10A3, Santa Cruz Biotechnology, Dallas, TX, USA). Next, they were incubated with appropriate secondary antibodies (anti-rabbit and anti-mouse from Thermo Fischer Scientific, Waltham, MA, USA) conjugated to peroxidase. The bands were detected using the enhanced Westar Supernova chemiluminescence assay (SuperSignal Westar SuperNova Chemiluminiscent Substrate, Thermo Fischer Scientific, and Waltham, MA, USA) and CL-XPosureTM (X-Ray) films (Thermo Fischer Scientific, Waltham, MA, USA). The films were scanned and the resulting images were analyzed by densitometry to determine the relative levels of each protein, using the Un-Scan-IT gel 6.1 software.

### RNA Extraction and qPCR

Total RNAs were extracted from primary mouse neuronal cultures (plated at 1 × 10^6^ cells/cm^2^ on 35 mm dishes) using the Qiagen RNA extraction kit at different times after infection with HSV-1 (1, 4, 8, 24 hpi). cDNAs were generated using the First-Strand cDNA synthesis kit (Invitrogen, Carlsbad, CA, USA) and qPCR was performed using SYBR green PCR Master Mix (Agilent Technologies, Santa Clara, CA, USA) using the following forward and reverse primers: PPIAm: GAG CAC TGG GGA GAA AGG AT, CTT GCC ATC CAG CCA CTC AG; ARCm: CAC TCT CCC GTG AAG CCA TT, TCC TCC TCA GCG TCC ACA TA; ICP8. qPCR was performed using SYBR green PCR Master Mix (Agilent Technologies, Santa Clara, CA, USA) using the following forward and reverse primers: UL30: AGA GGG ACA TCC AGG ACT TTG T, CAG GCG CTTGTT GGT GTA C.

### Preparation of shRNA Lentiviral Particles

We generated stable H4 neuroglioma cell lines with reduced levels of Arc by introducing shRNAs with lentiviral particles. The shRNA sequences were cloned in the pLKO.1 vector obtained from Sigma-Aldrich (NM-015193). An shRNA against the luciferase gene was employed as a control. Lentiviral particles were generated by co-transfection of HEK293 cells with the pLKO.1-shRNA constructs (1 μg), VSV-g (1 μg) and pΔ8.9 (1 μg). Transfections were performed with Lipofectamine 2000 (Invitrogen, Carlsbad, CA, USA) following the manufacturer’s instructions. Forty-eight hours post-transfection, media with lentiviral particles were transferred to H4 cells in a 1:2 dilution in the presence of 8 μg/ml polybrene. After 24 h, cells were selected with 3 μg/ml of puromycin and knockdown (KD) cells confirmed by Western blotting.

### Statistical Analysis

All the results are representative of at least three independent experiments. Results were analyzed by one-way or two-way analyses of variance (ANOVA) using the GraphPad Prism v.6 program. The data were expressed as means ± standard deviations. The *p* values were reported in each case; *p* < 0.05 was considered significant.

## Results

### Arc Protein Levels in Brain Sections of HSV-1-Infected Mice

**Figure 1:**
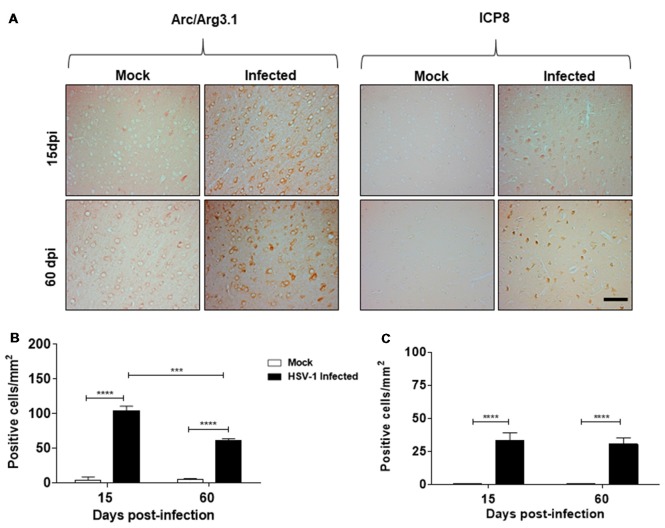
Herpes simplex virus type 1 (HSV-1)-infected mice present elevated levels of Arc protein.** (A)** Cortexes from mock and HSV-1 infected mice (six animals per time and condition) were stained at 15, 60 days post-infection (dpi) post-infection with antibodies to Arc and to ICP8 (three slices per animal). Magnification 100×, scale bar represents 100 nm. **(B,C)** Graphics show the number of Arc/ICP8 positive cells/mm^2^ (*n* = 3 sections per times). ****p < 0.0001 and ***p < 0.001 [analyzed by Two-Way analyses of variance (ANOVA)]. Statistical analyses between mock times to Arc and ICP8 were not significant. Also were not significant to ICP8 in infected times at 15 dpi compared to 60 dpi.

In this study, we sought to elucidate whether neurotropic HSV-1 infection could have a role in Arc protein expression, a protein highly implicated in synaptic plasticity. For this purpose, we first examined paraffin-embedded cortex sections from mock and HSV-1 infected mice generated previously by Martin et al. (2014a). Mice were nasally inoculated with HSV-1 strain F (2 × 10^5^ PFU/mice) and euthanized at 15 and 60 dpi. Sections of cortical brain areas were analyzed using anti-Arc and anti-ICP8 antibodies. Figure 1A shows an intense immunoreaction of Arc protein and ICP8 viral marker in serial sections of cortical brain areas under HSV-1 infection in comparison to mock infected mice, evidencing that HSV-1-infection triggers an increase in Arc protein levels. Although the highest levels were detected during acute infection at 15 dpi, we found that increased levels persisted even after disappearance of the symptoms at 60 dpi (Martin et al., 2014a). These findings strongly suggest that HSV-1 *in vivo* infection causes a recurrent Arc effect during subclinical reactivations (see Supplementary Figure S2). Graphic representation (Figures 1B,C) shows a significant number of Arc and ICP8 positive cells/mm^2^ after infection compared to mock infected samples.

### HSV-1 Neuronal Infection and Replication Increases mRNA and Protein Levels of Arc

Four types of neuronal cultures, H4 human neuroglioma, SH-SY5Y human neuroblastoma and HT22 mouse hippocampal neuroblastoma cultures, were used to carry out the majority of the analyses; however, taking into consideration the international animal care and experimentation recommendations^2^, primary mouse neuronal cultures were used only in some experiments to obtain a more physiological approach.

**Figure 2:**
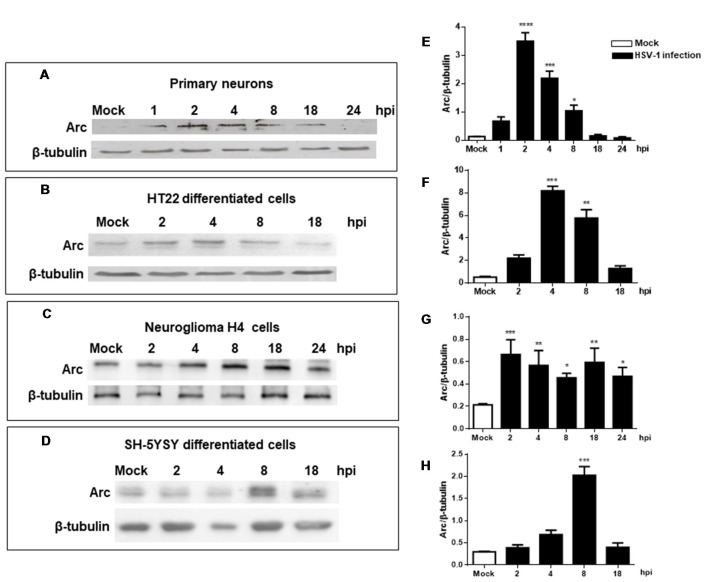
Increased levels of Arc protein during *in vitro* neuronal HSV-1 infection. Immunoblot analyses showing Arc and β-tubulin proteins, in either non-infected (Mock or M) or HSV1-infected brain cells multiplicity of infection (MOI = 10). **(A)** Mouse primary culture neurons. **(B)** Differentiated HT22 cells. **(C)** Neuroglioma H4 cells. **(D)** Differentiated SH-SY5Y cells. Infections were performed at 1, 2, 4, 8, 18 and 24 hours post-infection (hpi). **(E–H)** After gel scanning and densitometry quantitation, the results were expressed as the ratio of Arc protein levels respect to total tubulin. The blots are representative of three independent experiments. ****p < 0.0001; ***p < 0.001; **p < 0.01; *p < 0.05 (analyzed by One-Way ANOVA, followed by Tukey’s multiple comparisons Test).

Our findings were confirmed analyzing levels of Arc protein during HSV-1 infection in primary neuronal cultures and different brain cell types, including differentiated HT22 and SH-SY5Y (see Supplementary Figure S1) and H4 cells. Figure 2 shows that all cells tested including primary culture neurons showed high levels of Arc protein during HSV-1 infection. Although this is a common phenotype, we observed certain differences with respect to the timing and magnitude of the response in the cell types analyzed. For instance, we observed a rapid increase in Arc levels during the first 2 h of infection in primary culture neurons and HT22 cells. In contrast, we found that in H4 and SH-5YSY cells, Arc levels increased greatly during the 8 h of infection. Regardless of the different timings observed, we found that in all cases the increase in Arc protein levels was transitory, observing a consistent decay in Arc protein after 8 h of HSV-1 infection.

**Figure 3:**
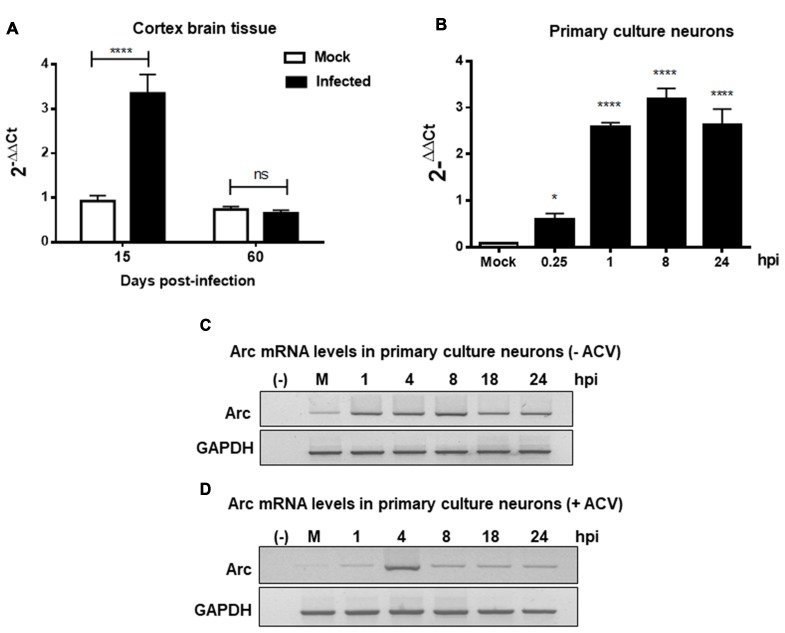
HSV-1 neuronal infection and replication triggers upregulation of Arc mRNA levels. Arc mRNA levels were analyzed by RT-qPCR in either **(A)** Brain cortex of mock and HSV-1 infected mice at 15 or 60 dpi or **(B)** mouse primary culture neurons infected during 24 h with HSV-1 and mock conditions. Relative Arc mRNA expression levels were controlled in respect to the levels of the housekeeping gene PPIA. After gel scanning and densitometry quantitation, the results were expressed as the ratio of Arc mRNA in respect to PPIA mRNA levels. Experiments were performed in triplicate. ****p > 0.0001 and *p > 0.05; ns: non-significant (using Two-Way ANOVA analyze in graphic **A**, and One-Way ANOVA analyze in graphic **B**). Conventional RT-PCR in Mock- and HSV-1-infected (MOI = 10) mouse primary culture neurons during 1, 4, 8, 18 and 24 hpi without **(C)** or with **(D)** acyclovir (ACV) treatment added during the time course of infection.

**Figure 4:**
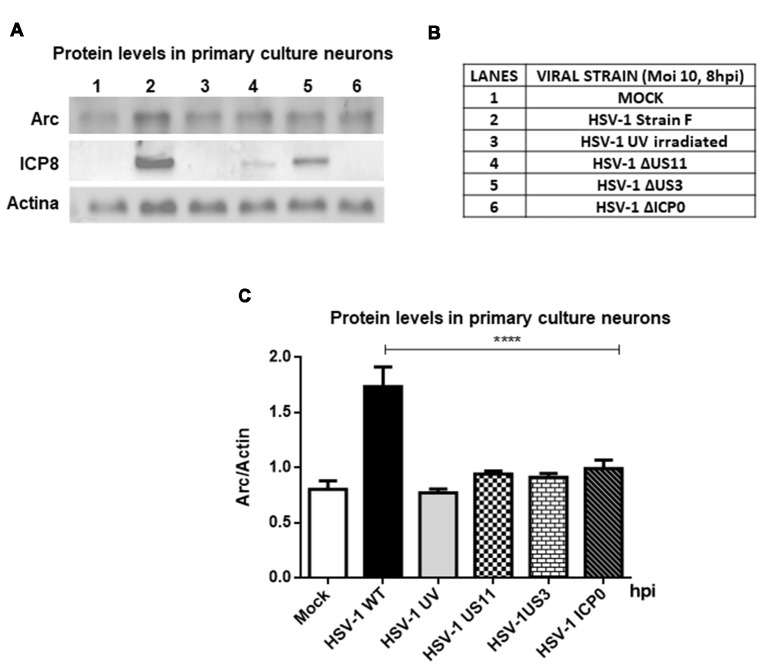
Viral replication is mandatory to induce an increase in Arc protein levels.** (A)** Immunoblot analyses showing Arc, ICP8, Actin and GAPDH proteins, in either mock or HSV-1-infected neurons (lanes 1–6) with a MOI of 10 and at 8 hpi. **(B)** Table indicating the strains tested including Strain F (wild-type HSV-1) and different replicative-defective HSV-1 mutants and UV-irradiated (inactivated) HSV-1. **(C)** After gel scanning and densitometry quantitation, the results were expressed as the ratio of Arc protein levels in respect to total Actin. The results shown are representative of three independent experiments. ****p > 0.0001 (analyzed by one-way ANOVA, followed by Tukey’s multiple comparisons test).

Furthermore, to evaluate whether high Arc protein levels could be the result of a positive transcriptional regulation, RT-qPCR from brain cortex samples of 15 and 60 dpi were performed. We observed a strong increase in Arc mRNA levels in cortex brain tissue after 15 days, confirming transcriptional upregulation of Arc under HSV-1 infection. In contrast, Arc mRNA levels in 60 dpi were unaltered (Figure 3A). Considering that Arc protein levels were greatly increased in 60 dpi, this result strongly suggests that Arc protein is stabilized in infected neurons in a long-term fashion. Consistent with these findings, we observed that *in vitro* HSV-1 infection in primary neuronal cultures caused a sustained increase in Arc mRNA levels during 24 h of infection (Figure 3B). To investigate whether the increase in Arc mRNA levels was the result of HSV-1 replication we used ACV, a well-known inhibitor of HSV-1 replication. Primary culture neurons were infected with HSV-1 (MOI = 10), in the absence or presence of ACV, and the cells were analyzed by conventional RT-PCR analysis. We observed a sustained increased in Arc mRNA levels during 24 h of HSV-1 infection (Figure 3C). However, in the presence of ACV we observed that, in the initial increase of Arc mRNA levels at 4 h of HSV-1, the infection had a rapid rate of decay when compared to ACV-untreated cells (Figure 3D). This result suggests that an increase in Arc mRNA levels could be the result of an active replicative cycle. Furthermore, to confirm the role of HSV-1 viral replication in Arc protein levels, we tested different replicative-defective HSV-1 mutants and UV-irradiated (inactivated) HSV-1. Our results demonstrated that viral replication is mandatory to trigger an increase in Arc protein expression, observing that only HSV-1 Strain F (wild-type) was capable of inducing a robust increase in Arc protein levels (Figures 4A–C).

### Cellular Distribution of Arc Protein Upon HSV-1 Neuronal Infection

**Figure 5:**
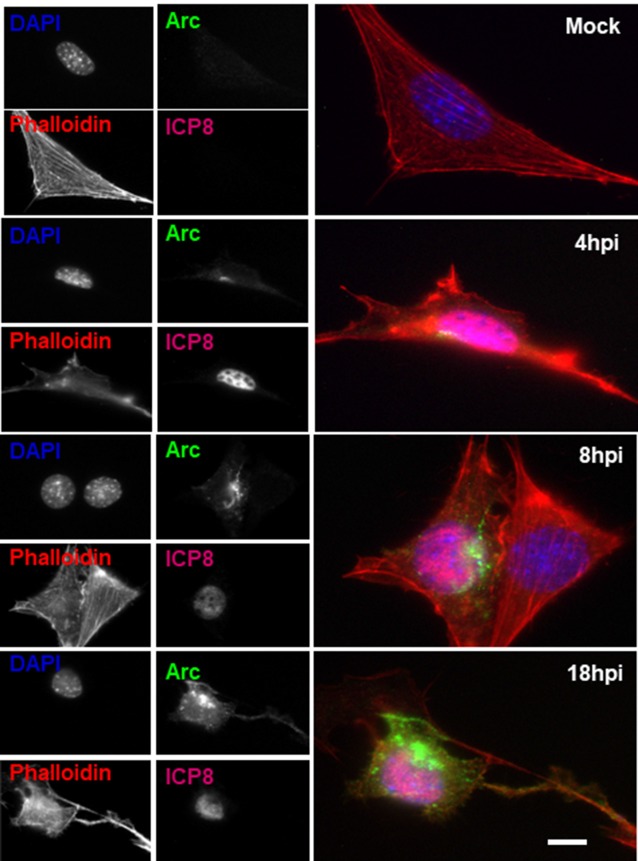
Altered Arc protein distribution during HSV-1 neuronal infection. Untreated (mock) or HSV-1-infected HT22 cells (MOI = 10) during 4, 8 or 18 hpi were stained with specific antibodies to Arc or to ICP8 followed by secondary antibodies coupled to Alexa Fluor 488 and 647 dyes, respectively. Phalloidin staining was coupled with Alexa Fluor 594 dye and Nuclei stained with DAPI. Scale bar corresponds to 10 μm. Magnification 630×. The results shown are representative of three independent experiments.

**Figure 6:**
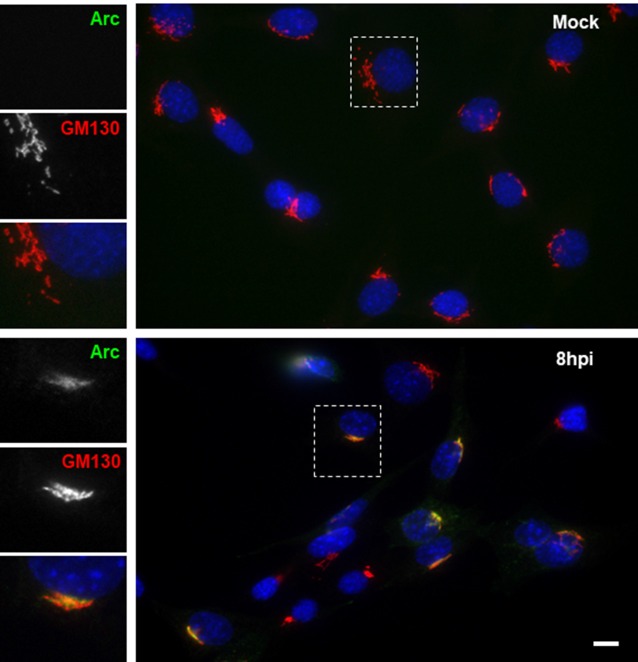
HSV-1 infection induced colocalization of Arc protein with the Golgi marker GM130 in HT22 cells. Untreated (mock) or HSV-1-infected (MOI = 10) HT22 differentiated cells during 8 hpi were stained with antibodies to Arc and the Golgi marker GM130 followed by secondary antibodies coupled to Alexa Fluor 488 and 594 dyes, respectively. Nuclei were stained with DAPI. Scale bar corresponds to 10 μm. Magnification 630×. The box inserted shows a zoom capture of the indicated area. The results shown are representative of three independent experiments.

To further examine the distribution of Arc upon HSV-1 infection, we performed immunofluorescence analysis using phalloidin staining to track cell shape changes during infection. Figure 5 shows ICP8 and Arc immunoreaction in differentiated HT22 neuronal cultures. We observed that in all infected cells, demonstrated by the expression of the viral marker ICP8 (nuclear pink signal), there was a positive reactivity to endogenous Arc and the protein that showed a perinuclear distribution. Phalloidin staining showed no changes in cellular shape after 8 h of HSV-1 infection compared to mock treated cells or cells infected for 4 or 18 h; we then selected 8 h of infection for further immunofluorescence analysis. Next, in order to study whether the perinuclear pattern of Arc could correspond to the Golgi apparatus, we studied colocalization with the Golgi marker GM130 during HSV-1 infection in differentiated HT22 neuronal cells. We found that Arc distributes with a similar pattern to the Golgi marker GM130 (see Figure 6 and Supplementary Figure S3), strongly suggesting that Arc is located at the Golgi apparatus during HSV-1 infection. As expected, in mock-infected cells the Arc immunoreaction signal was almost undetectable, confirming low levels of this protein in the absence of viral infection. This result that was confirmed by Western blot analysis in different cell types (Figure 2). Altogether, our findings demonstrate that HSV-1 infection induces intracellular expression of Arc protein in neuronal cells *in vitro* and *in vivo*.

### HSV-1 Infection Alters Distribution of AMPAR Receptor by an Arc-Independent Mechanism

**Figure 7:**
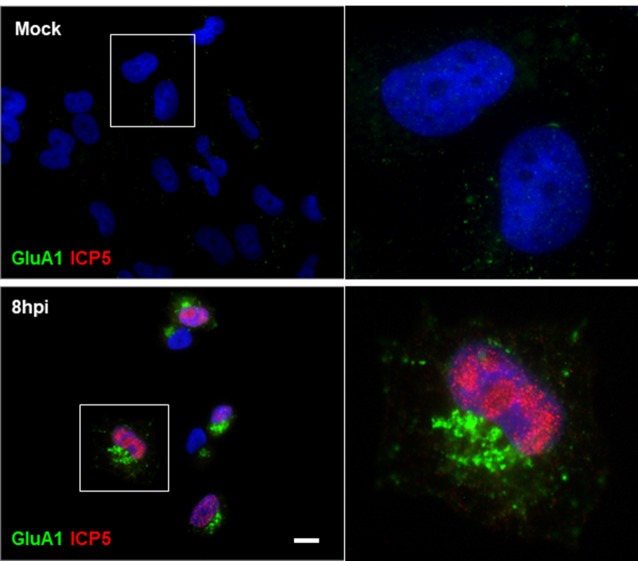
Redistribution of the AMPA receptor (AMPAR) GluA1 subunit during HSV-1 infection in H4 cells. Untreated (mock) or HSV-1-infected (MOI = 10) H4 cells during 8 hpi were stained with antibodies to GluA1 subunit and to ICP5 followed by secondary antibodies coupled to Alexa Fluor 488 and 594 dyes, respectively. Nuclei were stained with DAPI. Bar corresponds to 10 μm. Magnification 630×. The boxes inserted show a zoom capture of the indicated areas. The results shown are representative of three independent experiments.

**Figure 8:**
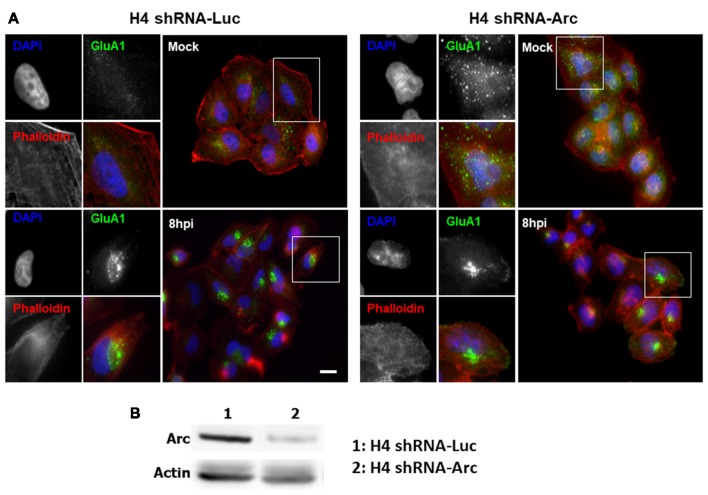
Redistribution of AMPAR GluA1 subunit during HSV-1 infection is independent of Arc expression.** (A)** H4 cells stably expressing either the shRNA to luciferase (H4 shRNA-Luc) used as a control or the shRNA to Arc (H4 shRNA-Arc) to achieve silencing of Arc knockdown (KD) were infected with HSV-1 (MOI = 10) during 8 hpi. Then, GluA1 subunit and ICP8 were immunodetected with specific antibodies followed of secondary antibodies couple to Alexa Fluor 488 and 647, respectively. Phalloidin staining was coupled with Alexa Fluor 594 dye and Nuclei stained with DAPI. Scale bar corresponds to 10 μm. Magnification 630×. The boxes inserted show a zoom capture of the indicated areas. **(B)** Total protein extracts of H4 shRNA-Luc and H4 shRNA-Arc were analyzed by immunoblot with antibodies to the proteins Arc and GADPH. The results shown are representative of three independent experiments.

Based on our results showing that HSV-1 infection causes a significant increase in Arc protein levels, we investigated whether Arc could be participating in AMPAR endocytosis during HSV-1 infection in neuronal cells. To evaluate this, we first studied the distribution of the AMPAR GluA1 subunit during HSV-1 infection in H4 cells controlled with the expression of ICP5, the major capsid protein of HSV-1. We observed that expression of GluA1 in H4 cells is very low. In contrast, and surprisingly, we found that under HSV-1 infection, endogenous GluA1 was highly concentrated in punctate structures located at the perinuclear region (Figure 7 and Supplementary Figure S4), suggesting possible involvement of Arc expression in AMPAR internalization under basal condition. Furthermore, we tested whether distribution of GluA1 under HSV-1 infection could be dependent on Arc expression. For this, we compared H4 cells stably expressing either the shRNA to Arc (shRNA-Arc) or the shRNA to luciferase as control (shRNA-Luc). Interestingly, and similar to previous results by Chowdhury et al. (2006), we observed that Arc KD increases the levels of GluA1 at the plasma membrane (Figure 8), confirming our cellular model. Unexpectedly we found that GluA1 had a similar distribution pattern under HSV-1 infection in H4-shRNA-Luc or H4-shRNA-Arc, observing that both cell types redistributed GluA1 to the perinuclear region, as we observed in H4 parental cells. These findings confirmed the role of Arc in the internalization of AMPARs under basal conditions and discarded the role of Arc in the redistribution of AMPARs during HSV-1 infection. Moreover, our results strongly suggest that HSV-1 infection alters synaptic function through changes in AMPAR distribution.

### Arc Enhances HSV-1 Viral Progeny *in vitro*

**Figure 9:**
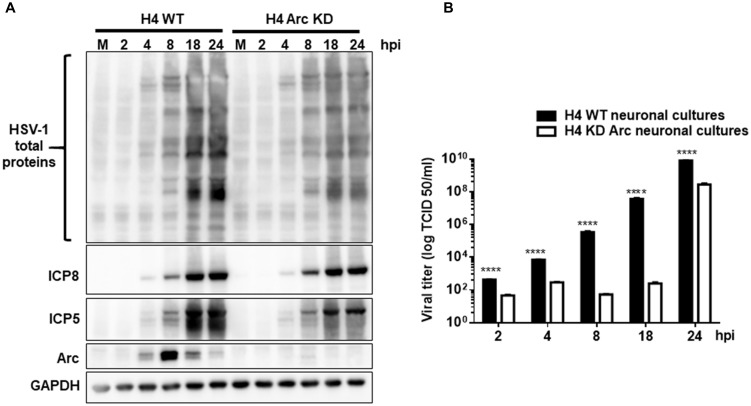
Arc enhances HSV-1 viral progeny *in vitro*. **(A)** H4 cells stably expressing either the shRNA to luciferase (H4 shRNA-Luc) used as a control or the shRNA to Arc (H4 shRNA-Arc) to achieve silencing of Arc KD were infected with HSV-1 (MOI = 10) during 2, 4, 8, 12, 18 y 24 hpi. First, infected cells were harvested and the production of infectious viral progeny in the supernatants measured in each time point by a standard TCID_50_ assay. Second, total protein extracts were analyzed by immunoblot with antibodies to the proteins ICP8, ICP5, Arc and GADPH. **(B)** Graphic shows the viral titer of HSV-1 in the supernatants from H4 shRNA-Luc (black bars) or H4 shRNA-Arc (white bars). The results shown are representative of three independent experiments (****p > 0.0001, analyzed by two-way ANOVA).

As our results demonstrated that HSV-1 infection increases Arc levels, we investigated whether Arc protein is necessary for HSV-1 viral progeny *in vitro*. Interestingly, it has been recently described that Arc protein has a retroviral origin, sharing structural similarities with the Gag retroviral capsid protein (Ashley et al., 2018; Pastuzyn et al., 2018). It is plausible then that expression of Arc in host cells could play a crucial role in HSV-1 progeny. Arc contained protein-protein interaction domains and has the property to form oligomers and a capsid-like structure, which is a feature of neurotropic viral infections. In order to elucidate whether HSV-1 requires Arc protein for its own replication and progeny, H4-shRNA-Luc or H4-shRNA-Arc were infected with HSV-1 during different times and compared to mock-infected cells. Following this, cell extracts were analyzed by Western blot and cell culture medium used in a viral infectivity TCID_50_ assay. We observed that Arc KD induced a significant decrease in the expression of viral antigens and in viral production (Figures 9A,B). Altogether, these findings indicate that Arc protein plays a crucial role in HSV-1 viral progeny.

## Discussion

The prevalence of dementia among individuals aged 71 and older is 13.9%; however, most individuals will develop milder cognitive deficits known as age-associated cognitive decline (Penner et al., 2010). The study of the mechanisms that contribute to age-associated cognitive decline will not only lead to a better general understanding of memory function, but provides hope for more effective prevention strategies and improvement of the quality of life for our aging population (Penner et al., 2010; Bearer, 2012; Kobayashi et al., 2013; Mancuso et al., 2014).

In recent years a number of studies have suggested that the intracellular accumulation of Aβ is an early hallmark of Alzheimer’s disease (AD), and that its oligomerization leads to synaptic dysfunction before neuronal death occurs (Li et al., 2007; Cappai and Barnham, 2008). However, neither the causes nor the molecular mechanisms behind the accumulation of Aβ in sporadic AD are well known.

On the other hand, previous studies have demonstrated that HSV-1 infects structures in the CNS, similar to those damaged in AD. Thus, HSV-1 represents a risk factor in the development of chronic neurological and neurodegenerative pathologies—especially related to aging—where repeated cycles of viral reactivation might contribute to generating progressive neuronal damage (Bearer, 2012; Otth et al., 2016; Itzhaki and Tabet, 2017). Among different clinical studies, a prospective study performed on persons over 65 years old over 12 years provided the strongest evidence of an association between HSV-1 infection and AD progression, considering the presence of anti-HSV IgM antibodies as individuals with viral reactivation episodes (Letenneur et al., 2008). In agreement with these studies, high level of HSV-1 antibodies in patients with AD are currently correlated with cortical atrophy of the gray matter confirmed through magnetic resonance (Mancuso et al., 2014) and cognitive impairment evaluated through clinical tests that evaluate mental capacities (MMSE; Kobayashi et al., 2013).

In fact, recent genetic studies support a model that links a specific gene network with AD and in close association with viruses, proposing the hypothesis that pathogens are the main contributing risk factor for AD pathogenesis (Readhead et al., 2018). This is in agreement with the studies that link HSV-1 with AD.

Moreover, it is well established that transcription of important IEGs, including the Arc gene, are selectively regulated by neural activity in several regions of the brain, including the hippocampus, key responses for synaptic plasticity and memory consolidation (Guzowski et al., 2000; Steward and Worley, 2001; Bramham, 2007; Bramham et al., 2008). This exquisite regulation suggests that Arc protein might play a crucial role in synaptic function and that any alteration in its levels may have direct consequences in brain function impairment (Shepherd and Bear, 2011; Soulé et al., 2012; Kerrigan and Randall, 2013). In this sense, our results revealed that HSV-1 could have an impact on synaptic plasticity and memory consolidation due to the increase in Arc protein levels. Interestingly, we demonstrated that the increase in Arc levels requires the replicative cycle of the virus, observing that none of the replicative-defective mutants of HSV-1 or the inactivated HSV-1 (UV-irradiated) had an effect on Arc. Due of these findings, we propose that accumulative reactivation episodes of HSV-1 in SNC must be triggering neuronal dysfunction through a dysregulation in Arc neuronal protein expression.

Mechanistically, Arc protein plays a crucial role in the trafficking of AMPA neuronal receptors, key players for neuronal plasticity and excitability. Arc directly binds endocytic machineries, accelerating the removal of AMPARs from excitatory synapses (Chowdhury et al., 2006; Penner et al., 2010; Wu et al., 2011). In fact, in Arc KO mice, the endocytosis of AMPARs was dramatically reduced, observing an enhanced steady-state distribution at the plasma membrane (Chowdhury et al., 2006). In agreement with this, our results showed that silencing of Arc in H4 cells resulted in an increased signal of GluA1 at the plasma membrane. Despite the inhibition in GluA1 internalization in Arc KD cells, we observed that GluA1 was located intracellularly under HSV-1 infection, suggesting that HSV-1 might overcome this inhibition in internalization, activating an alternative trafficking route.

Localization of Arc at the Golgi apparatus under HSV-1 infection could represent a site for viral assembly and viral progeny. Recently, Martin et al. (2017) reported that during HSV-1 neuronal infection the virus triggers Golgi fragmentation through Src-dynamin-2 activation to facilitate viral exocytosis processes necessary for viral liberation. Interestingly, Arc contains binding sites for endophilin-3 and dynamin-2, two proteins involved in the internalization of AMPARs (Byers et al., 2015). These findings open the possibility that the interactome of Arc at the Golgi apparatus could play a pivotal role in the release of HSV-1 particles at the Golgi apparatus in favor of its progeny.

On the other hand, Arc is a candidate in the regulation of Aβ generation at endocytic compartments (Huse et al., 2000). Arc has been described as a key player in the trafficking of the γ-secretase complex through endosomes in neurites, interacting directly with the N-terminus of presenilin 1 (PS-1), one of the four core proteins in the presenilin complex (Wu et al., 2011). This complex mediates the proteolytic processing of several transmembrane proteins, including the cleavage of the amyloidogenic C-terminal membrane fragment C99, the direct precursor of Aβ (Bustamante et al., 2013). Disrupted interaction between Arc and PS-1 prevents Aβ generation, supporting a pathogenic role of Arc in neurons (Wu et al., 2011). Interestingly, AD patients express abnormally high levels of Arc protein. In agreement with this, primary neurons and glial cells infected with HSV-1 lead to a dramatic increase in extracellular Aβ deposits (Wozniak et al., 2007) and intracellular Aβ species (De Chiara et al., 2010). Our findings strongly suggest that HSV-1 infection and the consequent elevation in Arc protein levels could contribute to explaining the increased levels in intracellular and extracellular Aβ species found in AD patients.

High levels of Arc have also been implicated with microglia activation, suggesting that Arc could contribute to neuroinflammation, a scenario that is known to perturb neuronal plasticity and memory consolidation (Rosi et al., 2005) and cognitive deficits in AD. Thus, Arc emerges as an attractive player of synaptic dysfunction (Kawashima et al., 2009; Kerrigan and Randall, 2013) and as a positive regulator of Aβ production (Wu et al., 2011).

Our study is the first report that links HSV-1 with Arc neuronal protein. Our findings strongly suggest that pathogenicity of HSV-1 neuronal reactivations in humans could be mediated in part by Arc neuronal upregulation. Moreover, we present evidence that demonstrates the role of Arc in HSV-1 progeny and we postulate a possible intracellular niche for viral replication and release at the level of the Golgi apparatus. We propose that Arc mediates some of the pathogenic roles of HSV-1 through the impairment in AMPARs intracellular trafficking. Moreover, we discuss other alternatives, including the increase in Aβ production and its potential role in microglia activation and neuroinflamation (Supplementary Figure S5 shows level of Arc protein and mRNA during HSV-1 infection in primary astrocytes cultures).

## Author Contributions

CO and MC: conceived and designed the experiments. FA-H, MM, EM, MH, CM, LL and PS performed the experiments. CO, MC, PB, AZ and FA-H analyzed the data. MC, PB, AZ and CO contributed reagents, materials and analysis tools. CO, MC, AZ, PB and FA-H wrote the article.

## Conflict of Interest Statement

The authors declare that the research was conducted in the absence of any commercial or financial relationships that could be construed as a potential conflict of interest.

## Footnotes

^1^http://www.lal.org.uk

^2^http://www.lal.org.uk
