# Supplementary figures and images for: Herpes Simplex Virus Type 1 Enhances Expression of the Synaptic Protein Arc for Its Own Benefit
Source: Front Cell Neurosci. 2019 Jan 8;12:505. doi: 10.3389/fncel.2018.00505 (PMC6340317; doi:10.3389/fncel.2018.00505)

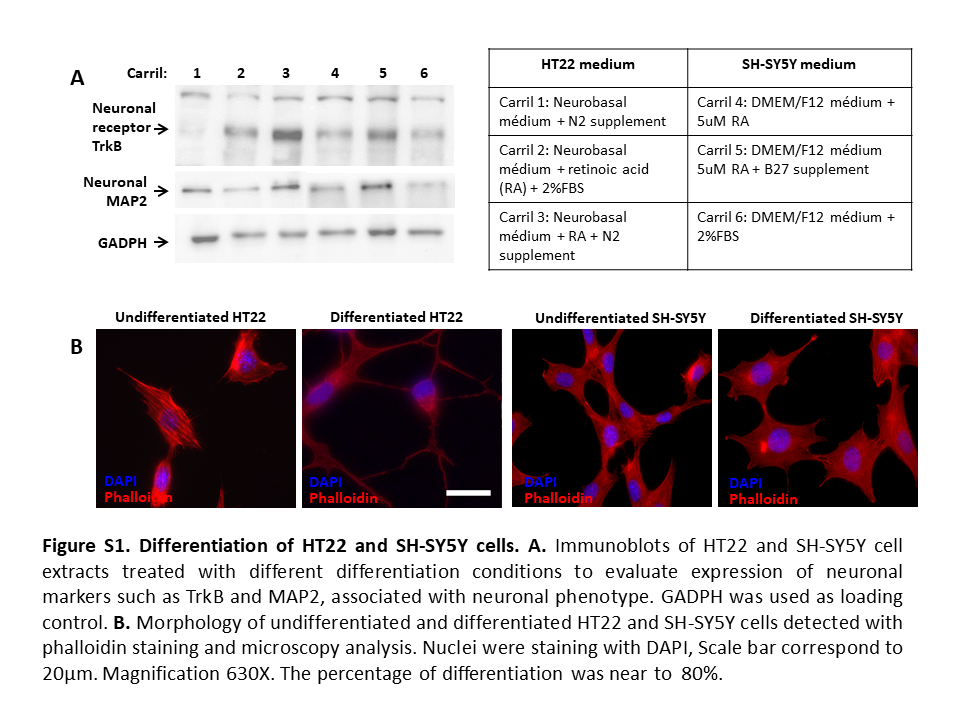

Supplement: Supplementary file 1 [file Image_1.TIF]

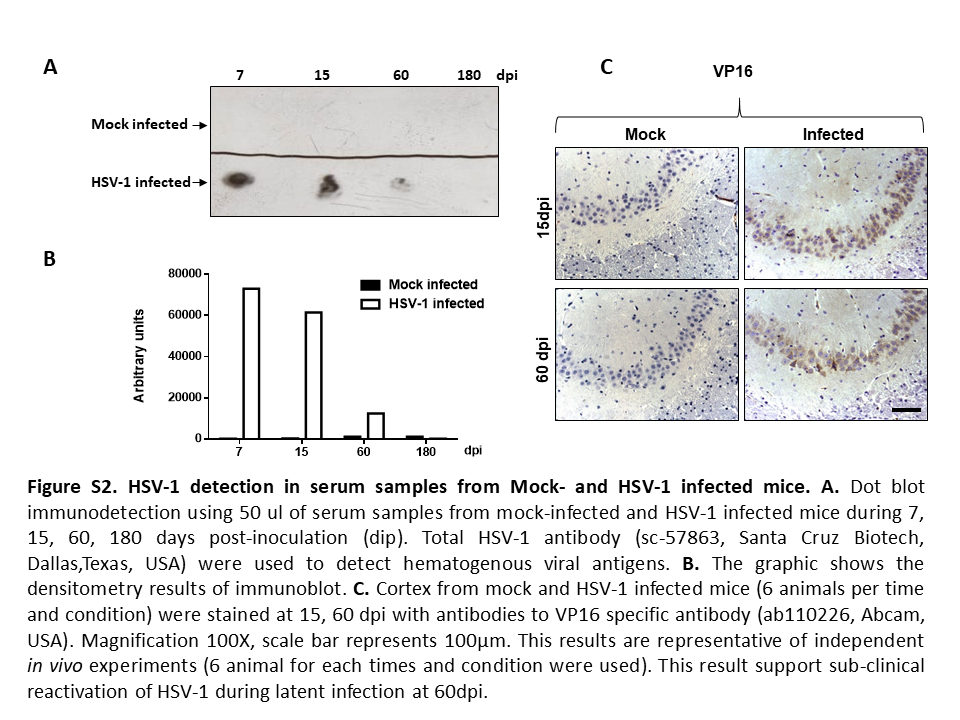

Supplement: Supplementary file 2 [file Image_2.TIF]

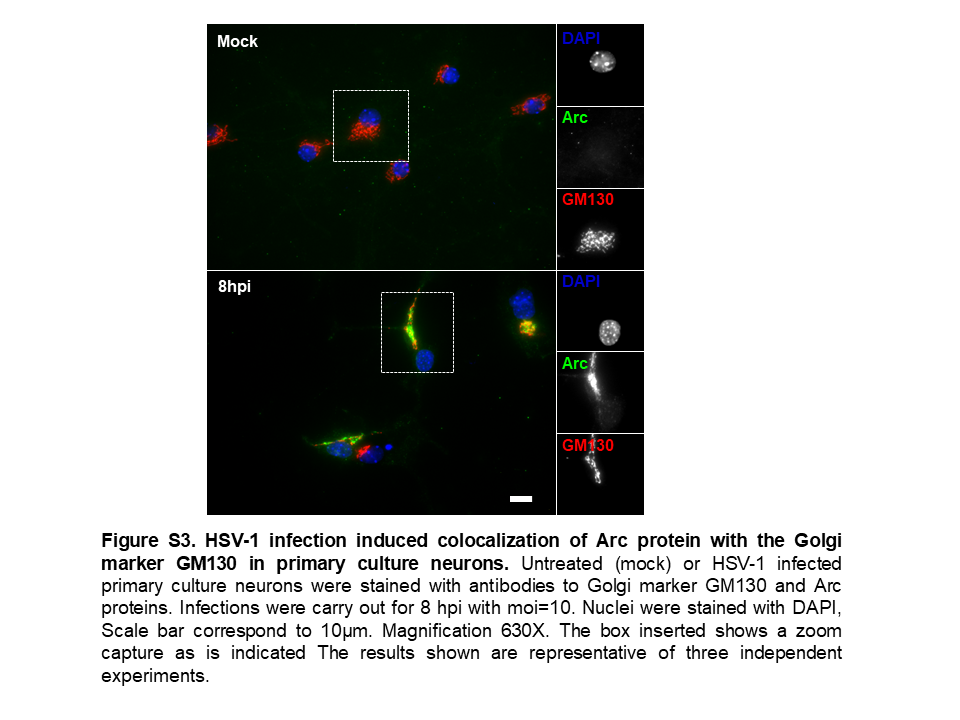

Supplement: Supplementary file 3 [file Image_3.TIF]

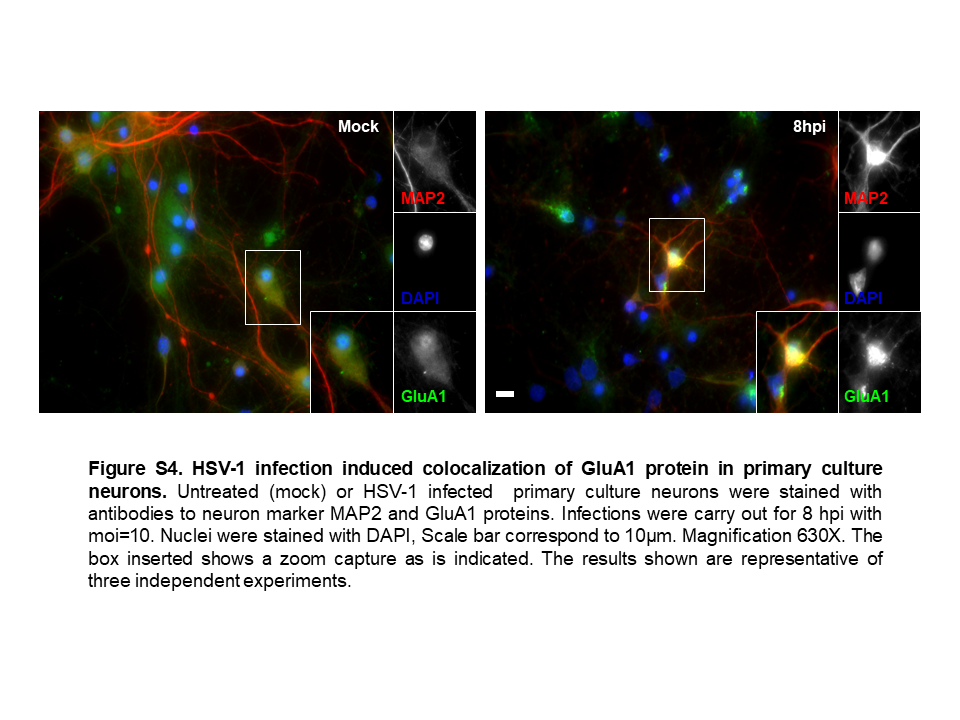

Supplement: Supplementary file 4 [file Image_4.TIF]

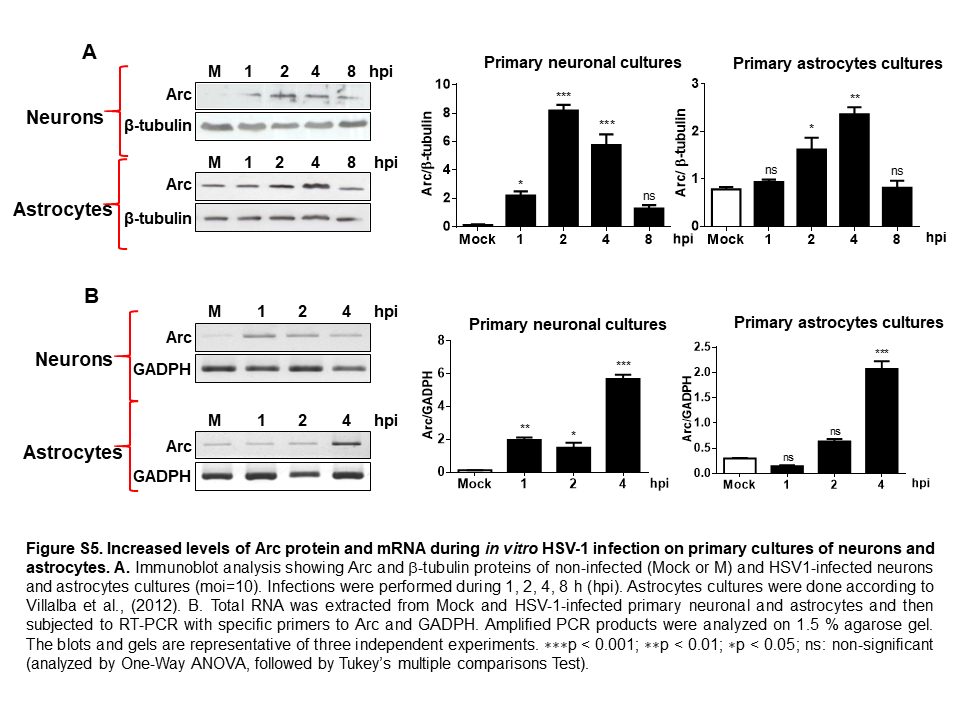

Supplement: Supplementary file 5 [file Image_5.tif]
